# Hepatic SIRT6 protects against cholestatic liver disease primarily *via* inhibiting bile acid synthesis

**DOI:** 10.7555/JBR.38.20240172

**Published:** 2024-12-02

**Authors:** Wen Zhang, Jiahui Wang, Luyao Yang, Yuyun Shao, Hongjun Peng, Longfeng Jiang, Liang Sheng

**Affiliations:** 1 Department of Pharmacology, School of Basic Medical Sciences, Nanjing Medical University, Nanjing, Jiangsu 211166, China; 2 Endoscopy Center, the First Affiliated Hospital of Nanjing Medical University, Nanjing, Jiangsu 210029, China; 3 Department of Pediatrics, Nanjing Drum Tower Hospital, Clinical College of Nanjing Medical University, Nanjing, Jiangsu 210008, China; 4 Department of Infectious Diseases, the First Affiliated Hospital of Nanjing Medical University, Nanjing, Jiangsu 210029, China

**Keywords:** sirtuin 6, cholestatic liver disease, oxidative stress, mitochondrial biogenesis, bile acid synthesis

## Abstract

Cholestatic liver disease, caused by the accumulation of hazardous bile acids in the liver, may result in cirrhosis, fibrosis, or liver failure. Activation of sirtuin 6 (SIRT6) prevents cholestasis-associated pathological events, such as oxidative stress and mitochondrial biogenesis dysfunction, and inhibits bile acid synthesis to alleviate cholestatic liver injury. However, it remains uncertain which pathway mediates the therapeutic effect of SIRT6 in reducing cholestasis. Therefore, we treated liver-specific *Sirt6* knockout mice with N-acetylcysteine, KEAP1-NRF2-IN-1, or acadesine to alleviate oxidative stress and/or promote mitochondrial biogenesis after modeling cholestatic liver disease, but these measures did not significantly improve cholestatic symptoms. However, MDL801, a SIRT6 agonist that downregulates cholesterol 7α-hydroxylase (CYP7A1, the key enzyme in bile acid synthesis) levels, exhibited favorable therapeutic effects. Additionally, the hepatic knockdown of *Cyp7a1* further demonstrated that inhibiting hepatic bile acid synthesis might be the main pathway through which SIRT6 alleviates cholestatic liver disease. These findings provide a solid basis for the potential application of SIRT6 agonists in treating cholestatic liver disease.

## Introduction

Cholestasis is a disorder involving bile stagnation and accumulation of toxic bile acids in the liver and bloodstream. It may arise from genetic and developmental abnormalities such as biliary atresia, as well as acquired diseases like those caused by viral hepatitis, drugs, metabolic syndrome, primary sclerosing cholangitis, primary biliary cholangitis, alcoholic liver disease, and obstruction by tumors or gallstones^[1]^.

Bile acids, particularly those with hydrophobic properties, are highly destructive to cells. Bile acids invade cellular mitochondria, thereby inhibiting the activity of the mitochondrial respiratory chain complex and ultimately causing an obstruction to the electron transfer in the respiratory chain. On the one hand, this increases the production of reactive oxygen species in hepatocytes and induces oxidative stress; on the other hand, it leads to adenosine triphosphate (ATP) depletion, disrupts mitochondrial membrane potential, increases mitochondrial permeability, and induces cell death. Mitochondrial DNA (mtDNA) encodes 13 key subunits of the respiratory chain complex and is highly susceptible to damage by oxidative stress. mtDNA damage leads to impaired expression of the respiratory chain complex, further exacerbating oxidative stress and mitochondrial dysfunction^[2–6]^. Persistent oxidative stress and mitochondrial dysfunction activate inflammatory signaling pathways in hepatocytes, leading to the release of cytokines and chemokines that further recruit immune cells to infiltrate the liver and create a chronic inflammatory state^[7]^. Inflammation and cellular damage activate hepatic stellate cells and promote collagen deposition, leading to alterations in liver structure and further deterioration of liver function^[8]^. Therefore, without timely treatment, chronic cholestasis may result in cirrhosis, fibrosis, and ultimately liver failure requiring a liver transplant^[9]^.

Sirtuin 6 (SIRT6), a member of the sirtuin family, possesses nicotinamide adenine dinucleotide-dependent deacetylase, mono-adenosine diphosphate-ribosyltransferase, and long-chain fatty acyl deacylase activities^[10–11]^, and regulates a variety of biological functions, including oxidative stress, inflammation, and mitochondrial biology^[12]^, which are involved in the development of cholestatic liver disease. It has been reported that SIRT6 counteracts oxidative stress by activating nuclear factor erythroid 2-related factor 2 (NRF2), which mediates the transcription of a range of antioxidant genes^[13–16]^. SIRT6 also activates AMP-activated protein kinase (AMPK), which in turn activates the transcriptional activity of peroxisome proliferator-activated receptor gamma coactivator-1α (PGC-1α) through phosphorylation modification and enhances the action of PGC-1α on its own promoter, thereby accelerating the expression of PGC-1α. As a regulator of mitochondrial biogenesis, PGC-1α enables SIRT6 to upregulate mitochondrial numbers and promote mitochondrial function^[17–19]^. Additionally, SIRT6 inhibits the estrogen-related receptor γ (ERRγ)-induced transcription of cholesterol 7 alpha-hydroxylase (CYP7A1), a key enzyme for bile acid synthesis, through deacetylation of ERRγ, thereby potentially attenuating bile acid accumulation and cholestasis^[20]^.

To identify the pathway through which SIRT6 exerts its therapeutic effects in reducing cholestasis, we used N-acetylcysteine (NAC), KEAP1-NRF2-IN-1 (KNI-1), acadesine (AICAR), or adeno-associated virus to knock down *Cyp7a1* to treat hepatic *Sirt6*-deficient mice. NAC, a synthetic derivative of L-cysteine, acts as a precursor for glutathione synthesis and enhances the activities of superoxide dismutase, glutathione reductase, and glutathione peroxidase in hepatocytes by upregulating the supplemental glutathione level to resist oxidative stress^[21]^. KNI-1 disrupts the interaction between NRF2 and its inhibitory binding protein, KEAP1, thereby preventing the degradation of NRF2, activating the expression of antioxidant genes downstream of NRF2, and protecting cells from oxidative stress^[22]^. AICAR, an activator of AMPK, promotes mitochondrial biogenesis by upregulating PGC-1α expression^[23]^.

The current study aimed to elucidate the molecular mechanism by which SIRT6 alleviates cholestasis. We employed active small molecules and gene knockdown techniques to investigate how SIRT6 influences cholestasis, specifically focusing on its roles in managing oxidative stress, promoting mitochondrial biogenesis, and inhibiting bile acid synthesis in mice with hepatocyte-specific *Sirt6* deletion.

## Materials and methods

### Animals

All practices concerning animal husbandry and experimentation followed the applicable institutional and national guidelines and were officially approved by the Animal Ethical and Welfare Committee of Nanjing Medical University (Approval No. 1811047). Jackson Laboratory in Maine, USA, provided the mice with *Sirt6* exons flanked by loxP sites (*Sirt6*^f/f^) and albumin-Cre transgenic mice on a C57BL/6 background. To establish a hepatocyte-specific deficiency in *Sirt6* (*Sirt6*^Δhep^), *Sirt6*^f/f^ mice were bred with albumin-Cre mice as reported previously^[24]^, and *Sirt6*^f/f^ mice were used as the wild-type counterparts. Wild-type C57BL/6 mice were obtained from the Animal Core Facility of Nanjing Medical University in Nanjing, China.

Seven-week-old male mice were infected with either adenovirus carrying *Sirt6* (Ad-*Sirt6*) or control adenoviruses (Ad-Control) at a dose of 5 × 10^10^ viral particles (VP) per mouse through tail vein injection, and surgery was performed seven days afterward.

Five-week-old male mice were infected with adeno-associated virus delivering sh*Cyp7a1* or control adeno-associated virus (shControl) at a dosage of 2 × 10^11^ genomic copies (GC) per mouse through tail vein injection, and surgery was performed 21 days afterward.

MDL801 [100 mg/(kg·day); TargetMol, Shanghai, China], KNI-1 [40 mg/(kg·day); MCE, Shanghai, China], NAC [200 mg/(kg·day); Solarbio, Beijing, China], and AICAR [250 mg/(kg·day); MCE] were administered to mice intraperitoneally one day before bile duct ligation (BDL) surgery, and the administration continued once daily for six additional days post-BDL.

Male mice at the age of eight weeks were anesthetized before undergoing either sham surgery or BDL^[25]^. Briefly, a midline laparotomy was performed, and then the common bile duct was double-ligated with a 4-0 suture and transected between the ligations. The mice in the sham group received similar surgery except for the bile duct ligation and transection. After surgery, the mice were placed on a heating pad in a cage and heated with an infrared lamp until they were fully awake and active.

The survival rate was monitored for 22 days following the surgical procedure. Blood samples and liver tissues gathered six days post-surgery were preserved at −80 ℃.

The timeline of all mouse treatments is shown in ***Supplementary Fig. 1*** (available online).

### Packaging and purification of the virus

Adeno-associated virus serotype 8 (AAV8) delivering sh*Cyp7a1* and shControl were prepared by Applied Biological Materials Inc. (Richmond, Canada). The titers of sh*Cyp7a1* and shControl viruses were 1.54 × 10^12^ GC/mL and 1.95 × 10^12^ GC/mL, respectively. Ad-*Sirt6* and Ad-Control were purchased from Hanbio (Shanghai, China). The virus titers of Ad-*Sirt6* and Ad-Control were 1.07 × 10^12^ VP/mL and 1.34 × 10^12^ VP/mL, respectively.

### Hematoxylin-eosin (H&E) staining

Liver tissues were immersed in 4% paraformaldehyde for 48 h, embedded in paraffin, and carefully sectioned at 6 μm. The tissue samples underwent H&E staining using a staining kit from Beyotime Biotechnology (Shanghai, China). Representative images were captured using a Leica microscope (Solms, Germany). H&E staining images were quantified using ImageJ software to determine the necrosis area.

### Determination of serum alanine aminotransferase (ALT), alkaline phosphatase (ALP), and bile acid levels

We examined serum ALT and ALP activities to assess liver injury. Blood samples were collected and centrifuged at 1200 *g* for 10 min to obtain the serum. Serum ALT and ALP activities were measured using commercially available assay kits (Cat. #C009-2-1 and Cat. #A059-2-1, respectively, Nanjing Jiancheng Bioengineering Institute, Nanjing, China) according to the manufacturer's instructions. Serum total bile acid levels were measured using a bile acid colorimetric assay kit (Cat. #E-BC-K181-M, Elabscience, Wuhan, China) following the manufacturer's instructions.

### Measurement of malondialdehyde (MDA), ATP, reactive oxygen species (ROS), and cytochrome C (Cyt C) contents

Liver tissues were rinsed with phosphate-buffered saline (PBS) and then homogenized and sonicated in lysis buffer on ice. After sonication, the lysed tissues were centrifuged at 10000 *g* for 10 min to remove debris. The MDA and ATP levels in the liver tissues were measured using an MDA (Cat. #BC0025, Solarbio) and an ATP assay kit (Cat. #BC0305, Solarbio), respectively. The hepatic ROS levels were determined using a ROS kit (Cat. #E004-1-1, Nanjing Jiancheng Bioengineering Institute, China) according to the manufacturer's instructions. Cytosolic Cyt C content in the liver tissues was assayed using an enzyme-linked immunosorbent assay kit (Cat. #ab210575, Abcam, Minneapolis, MN, USA) and normalized to total liver protein content. Liver cytosol was isolated following the instructions of this kit.

### Western blotting

Total proteins from liver tissues were extracted according to our laboratory approach described previously^[24]^. Briefly, proteins were separated by SDS-PAGE, immunoblotted with the indicated primary antibodies followed by horseradish peroxidase-conjugated secondary antibodies (1∶5000; anti-mouse IgG, Cat. #6805020 and anti-rabbit IgG, Cat. #671280; Biosharp, Hefei, China), and visualized with chemiluminescent horseradish peroxidase substrate (Cat. #1619502; Millipore, Boston, MA, USA) using a Tanon-5200 Chemiluminescence Imager (Tanon, Shanghai, China). Antibodies against SIRT6 (1∶1000; Cat. #12486), FLAG (1∶5000; Cat. #2368), BAX (1∶1000; Cat. #2772), cleaved caspase-3 (1∶1000; Cat. #9664s), H3 (1∶1000; Cat. #4499), AC-H3K9 (1∶1000; Cat. #9649), AMPKα (1∶1000; Cat. #5831), and p-AMPKα (Thr172) (1∶1000; Cat. #2535) were purchased from Cell Signaling Technology (Danvers, MA, USA), NRF2 (1∶1000; Cat. #16396-1-AP) and tubulin (1∶1000; Cat. #10068-1-AP) from Proteintech (Wuhan, China), CYP7A1 (1∶1000; Cat. #sc-518007) from Santa Cruz Biotechnology (Los Angeles, CA, USA), and PGC-1α (1∶1000; Cat. #ab54481) from Abcam.

### Quantitative reverse transcription-PCR (qRT-PCR)

Total RNAs of hepatocytes and livers were extracted using Trizol Reagent (Takara, Shiga, Japan). The StepOnePlus Real-Time PCR System (Applied Biosystems, Foster City, CA, USA) was used to perform qRT-PCR, using Thunderbird SYBR Master Mix (Toyobo, Osaka, Japan). Primer sequences were as follows: transcription factor A, mitochondrial (*Tfam*), 5*'*-ATTCCGAAGTGTTTTTCCAGCA-3*'* (forward), 5*'*-TCTGAAAGTTTTGCATCTGGGT-3*'* (reverse); heme oxygenase 1 (*Ho1*), 5*'*- AAGCCGAGAATGCTGAGTTCA-3*'* (forward), 5*'*-GCCGTGTAGATATGGTACAAGGA-3*'* (reverse); isocitrate dehydrogenase (NADP^+^) 2 (*Idh2*), 5*'*-GGAGAAGCCGGTAGTGGAGAT-3*'* (forward), 5*'*- GGTCTGGTCACGGTTTGGAA-3*'* (reverse); NAD(P)H quinone dehydrogenase 1 (*Nqo1*), 5*'*-AGGATGGGAGGTACTCGAATC-3*'* (forward), 5*'*-AGGCGTCCTTCCTTATATGCTA-3*'* (reverse); *36B4*, 5*'*-GGTCTGGTCACGGTTTGGAA-3*'* (forward) and 5*'*-CCGCAGGGGCAGCAGTGGT-3*'* (reverse).

### Measurement of mtDNA

We extracted mtDNA from liver tissues using the QIAamp DNA Mini kit (Qiagen, Valencia, CA, USA) and performed quantitative real-time PCR as described previously^[26]^. The *36B4* gene was used as a marker for nuclear DNA, and the cytochrome C oxidase 1 (*Cox1*) gene was used for mtDNA. Primer sequences were as follows: *Cox1*, 5′-TCTACTATTCGGAGCCTGAGC-3′ (forward) and 5′-CAAAAGCATGGGCAGTTACG-3′ (reverse); *36B4*, 5′-CGACCTGGAAGTCCAACTAC-3′ (forward) and 5′-ATCTGCTGCATCTGCTTG-3′ (reverse).

### Hepatocyte isolation and culture

Hepatocytes were isolated from mice receiving BDL or sham surgery according to a previous protocol^[27]^. Briefly, the mouse liver was perfused with Hank's balanced salt solution supplemented with 4 mmol/L NaOH, 10 mmol/L HEPES, and 0.5 mmol/L EGTA, followed by digestion with collagenase Ⅱ solution (0.6 mg/mL; Cat. #LS004177, Worthington Biochemical Corporation, Lakewood, NJ, USA). Hepatocytes were liberated from the liver, washed, and seeded in plates with William's E medium (Sigma-Aldrich, Shanghai, China) supplemented with 6% fetal bovine serum (Lonza, Richmond, VA, USA). The medium was changed after 2 h, and cells were cultured for another 16 h.

### Fluorescence MitoTracker Red staining

Mitochondrial staining was performed according to Ni *et al*^[28]^ with minor modifications. Briefly, hepatocytes were incubated with serum-free William's medium E containing 20 nmol/L MitoTracker (Cat. #C1035, Beyotime) in an incubator at 37 ℃ for 15 min. Cells were washed with PBS three times, and then incubated with 10 μg/mL Hoechst in serum-free William's E medium for 5 min. Following another PBS wash, high-magnification images of the mitochondrial morphology were captured using the Carl Zeiss LSM710 laser scanning confocal microscope.

### Statistical analysis

Data were presented as the mean ± standard error of the mean. Comparisons between the two groups were conducted using a two-tailed Student's *t*-test. One-way and two-way analysis of variance were used to compare data across more than two groups. *P* < 0.05 was considered statistically significant.

## Results

### *Sirt6* deficiency in the liver exacerbated cholestatic liver disease

**Figure 1:**
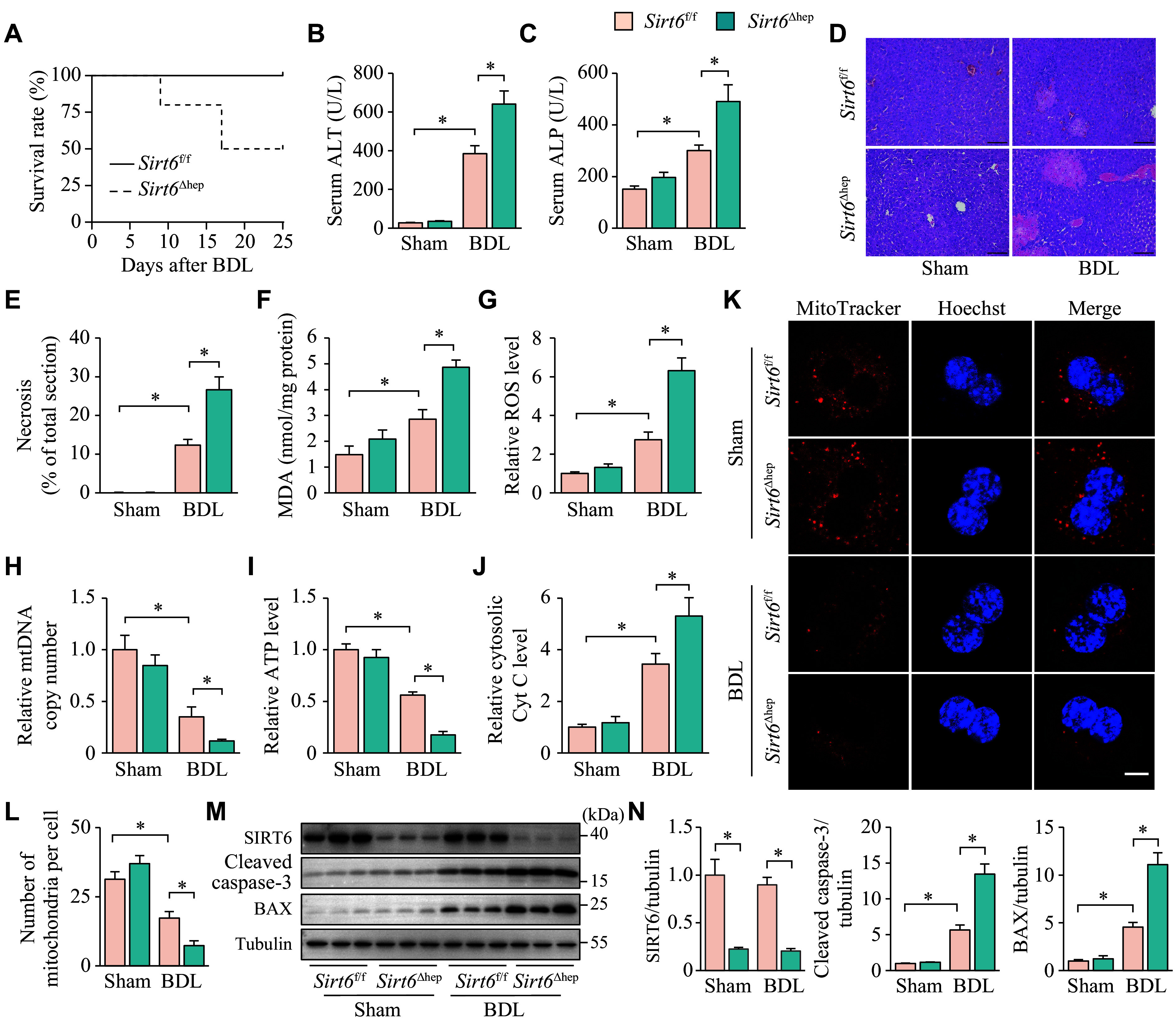
Hepatic *Sirt6* deficiency exacerbated cholestatic liver disease in mice. A: Survival curves of *Sirt6*^f/f^ (*n* = 10) and *Sirt6*^Δhep^ (*n* = 10) mice over 22 days post-BDL. B–N: *Sirt6*^f/f^ and *Sirt6*^Δhep^ mice underwent sham or BDL and were maintained for six days after surgery. These mice were divided into four groups: *Sirt6*^f/f^ + Sham, *Sirt6*^Δhep^ + Sham, *Sirt6*^f/f^ + BDL, and *Sirt6*^Δhep^ + BDL (*n* = 7 in each group). B and C: The serum was collected for ALT (B) and ALP (C) activity assays. D: The liver tissues were fixed in 4% paraformaldehyde and embedded in paraffin for hematoxylin-eosin staining. Scale bar, 100 μm. E: The ratio of necrotic area to total liver section area was calculated by ImageJ. F: The liver tissues were homogenized for the determination of MDA levels. G: The liver tissues were homogenized for the determination of ROS levels, which were normalized to the group *Sirt6*^f/f^ mice + Sham. H: mtDNA was extracted from the livers for quantitative PCR. *Cox1* was determined as an mtDNA marker with *36B4* as the internal standard. I: The liver tissues were homogenized for ATP level determination, which was normalized to the group *Sirt6*^f/f^ mice + Sham. J: Liver cytosol was extracted to assay cytosolic Cyt C levels, which were normalized to the group *Sirt6*^f/f^ mice + Sham. K: Hepatocytes isolated from mice underwent fluorescence MitoTracker Red staining (*n* = 3). Scale bar, 10 μm. L: The number of mitochondria was quantified from three different hepatocytes. M: The protein levels of SIRT6, cleaved caspase-3, and BAX in the liver tissues were determined by Western blotting (*n* = 3). Tubulin served as the internal control. N: The quantification of bands of SIRT6, cleaved caspase-3, and BAX. Data are presented as mean ± standard error of the mean and analyzed using two-way ANOVA followed by Tukey's honestly significant difference tests. ^*^P < 0.05. Abbreviations: BDL, bile duct ligation; ALT, aminotransferase; ALP, alkaline phosphatase; MDA, malondialdehyde; *Cox1*, cytochrome C oxidase 1; Cyt C, cytochrome C.

To investigate the role of SIRT6 in liver injury caused by cholestasis, we used mice with hepatocyte-specific deletion of *Sirt6* (*Sirt6*^Δhep^) and simulated extrahepatic cholestasis by BDL surgery^[25]^. We found that *Sirt6*^Δhep^ mice began to exhibit mortality nine days after BDL. By the 22nd day, their survival rate had fallen to 50%, in contrast to the control mice (*Sirt6*^f/f^) that showed no mortality during the same period (***Fig. 1A***).

We examined liver damage in both groups on the sixth day post-surgery, before the occurrence of death in either mouse group. As shown in ***Fig. 1B*** and ***1C***, compared with the *Sirt6*^f/f^ group, *Sirt6*^Δhep^ mice had a significant increase in serum ALT and ALP activities, which are indicators of injury to hepatic parenchymal cells and cholangiocytes. Moreover, the histological assessment indicated a significant expansion of the necrotic area in the livers of *Sirt6*^Δhep^ mice, compared with *Sirt6*^f/f^ mice (***Fig. 1D*** and ***1E***).

As oxidative stress and mitochondrial dysfunction are key pathological mechanisms in cholestatic liver disease^[3,29–30]^, we examined hepatic MDA and ROS levels to assess the degree of oxidative stress, and tested ATP levels to evaluate mitochondrial respiratory chain function. We also paid attention to mtDNA integrity because it houses the genetic information for respiratory chain proteins^[3,30]^. As shown in ***Fig. 1F***–***1I***, *Sirt6*^Δhep^ mice displayed significantly elevated levels of MDA and ROS in their livers, compared with *Sirt6*^f/f^ mice, accompanied by significant declines in mtDNA copy numbers and ATP levels.

Cyt C consists of proteins from the mitochondrial respiratory chain. When the mitochondrial structure is compromised, Cyt C is discharged from the mitochondria into the cytosol, activating the apoptotic pathway downstream. Hence, it plays a pivotal role as an indicator of both mitochondrial structural integrity and apoptosis^[31]^. After isolating the cytoplasm from mouse livers, we performed ELISA to assess the cytosolic Cyt C levels, and found that *Sirt6*^Δhep^ mice following surgery demonstrated higher concentrations of Cyt C in the liver cytoplasm, compared with *Sirt6*^f/f^ mice (***Fig. 1J***). The intracellular mitochondrial number, representing the combined result of mitochondrial damage and biogenesis, was detected by fluorescence MitoTracker Red staining. The mitochondrial number in hepatocytes was found to be significantly decreased after BDL. *Sirt6* deficiency itself did not affect the mitochondrial number, but in the presence of BDL, the mitochondrial number in *Sirt6*-deficient hepatocytes was significantly decreased, compared with those of the wild type (***Fig. 1K*** and ***1L***). Furthermore, Western blotting indicated a significant elevation in the hepatic levels of cleaved caspase-3 and BAX after BDL, two crucial proteins linked to apoptosis. Moreover, the deletion of *Sirt6* resulted in a further augmentation of their expression levels (***Fig. 1M*** and ***1N***).

Collectively, the *Sirt6*^f/f^ and *Sirt6*^Δhep^ mice that underwent sham surgery didn't show any significant variations in liver injury, oxidative stress, mitochondrial dysfunction, or apoptosis (***Fig. 1B***–***1N***). It appeared that *Sirt6* deficiency alone did not exhibit liver lesions. However, in the presence of cholestasis, *Sirt6* deletion intensified liver injury, oxidative stress, mitochondrial loss and dysfunction, and hepatocyte apoptosis.

### Hepatic overexpression of SIRT6 alleviated cholestatic liver disease

**Figure 2:**
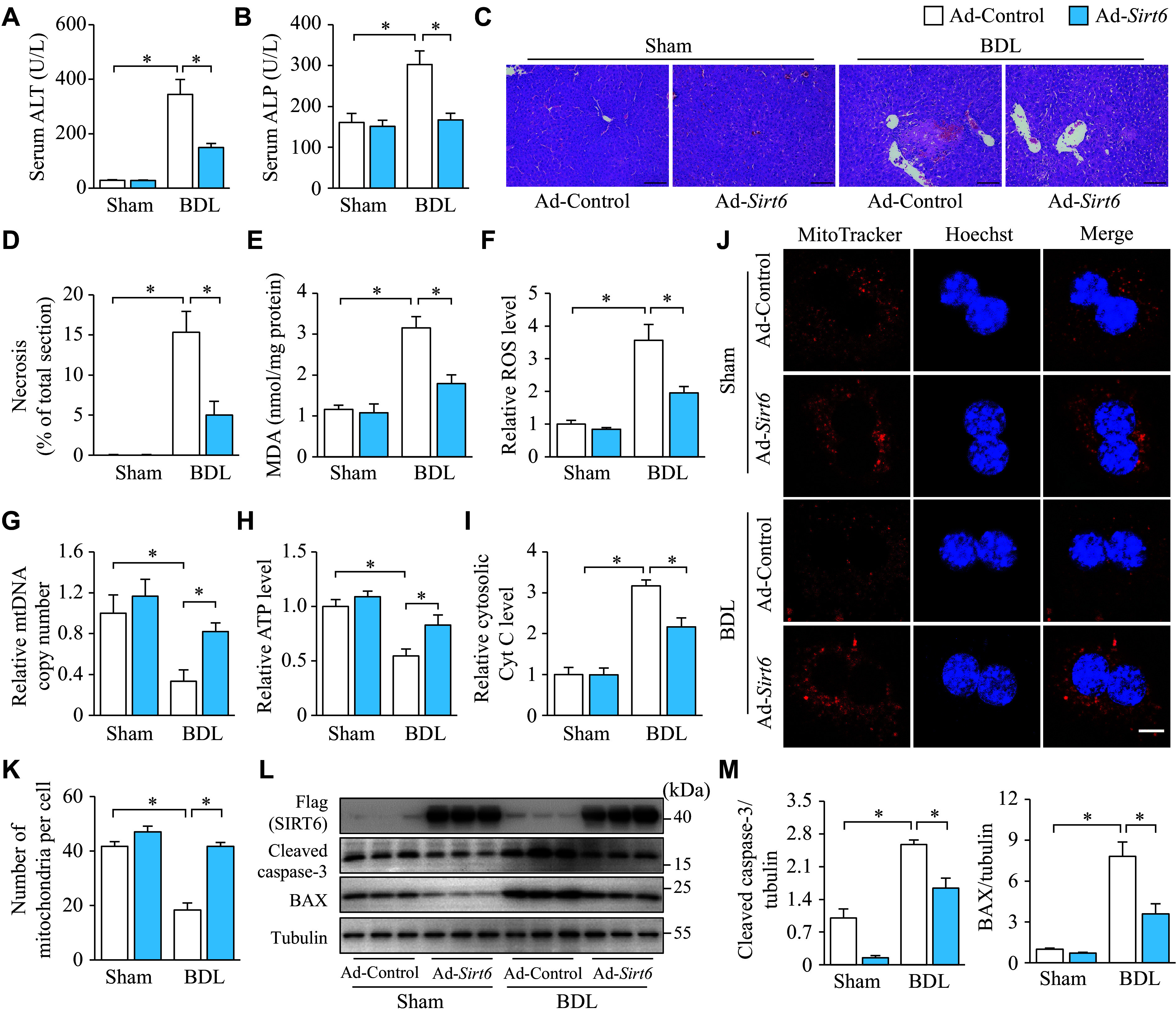
Hepatic overexpression of SIRT6 alleviated cholestatic liver disease in mice. C57BL/6 mice, infected with an adenoviral vector expressing SIRT6 (Ad-*Sirt6*) or control vector (Ad-Control), underwent sham or BDL seven days later and were maintained for six days after surgery. Mice were divided into four groups: Ad-Control + Sham, Ad-*Sirt6* + Sham, Ad-Control + BDL, and Ad-*Sirt6* + BDL (*n* = 7 in each group). A and B: The serum was collected for ALT (A) and ALP (B) activity assays. C: The liver tissues were fixed in 4% paraformaldehyde and embedded in paraffin for hematoxylin-eosin staining. Scale bar, 100 μm. D: The ratio of necrotic area to total liver section area was calculated by ImageJ. E: The liver tissues were homogenized to assay the MDA levels. F: The liver tissues were homogenized to assay the ROS levels, which were normalized to the group Ad-Control + Sham. G: mtDNA was extracted from the livers for quantitative PCR. *Cox1* was determined as an mtDNA marker with *36B4* as the internal standard. H and I: The liver tissues were homogenized to assay the ATP levels, which were normalized to the group Ad-Control + Sham. I: Liver cytosol was extracted to assay cytosolic Cyt C levels, which were normalized to the group Ad-Control + Sham. J: Hepatocytes isolated from mice underwent fluorescence MitoTracker Red staining (*n* = 3). Scale bar, 10 μm. K: The number of mitochondria was quantified from three different hepatocytes. L: The protein levels of SIRT6, cleaved caspase-3, and BAX in the liver tissues were determined by Western blotting (*n* = 3). Tubulin served as the internal control. M: The quantification of bands of SIRT6, cleaved caspase-3, and BAX. Data are presented as mean ± standard error of the mean and analyzed using two-way ANOVA followed by Tukey's honestly significant difference tests. ^*^P < 0.05. Abbreviations: BDL, bile duct ligation; ALT, aminotransferase; ALP, alkaline phosphatase; MDA, malondialdehyde; *Cox1*, cytochrome C oxidase 1; Cyt C, cytochrome C.

To further illustrate how SIRT6 functions in combating cholestatic liver disease, we used adenoviral vectors to overexpress SIRT6 in mouse liver, and subsequently carried out BDL. The results showed that hepatic overexpression of SIRT6 significantly reversed the BDL-induced effects, including the elevation in serum ALT and ALP levels (***Fig. 2A*** and ***2B***), the expansion of hepatic necrosis (***Fig. 2C*** and ***2D***), the increase in liver MDA and ROS levels (***Fig. 2E*** and ***2F***), and the reduction in mtDNA copy number and hepatic ATP levels (***Fig. 2G*** and ***2H***). Additionally, it prevented Cyt C release from mitochondria to the hepatocyte cytoplasm (***Fig. 2I***), reversed BDL-induced reduction in mitochondrial number (***Fig. 2J*** and ***2K***), and reduced the expression of cleaved caspase-3 and BAX proteins (***Fig. 2L*** and ***2M***). Under sham-operated conditions, SIRT6 overexpression in the liver did not show a significant influence on the above-mentioned aspects, and only slightly decreased the expression of cleaved caspase-3 and BAX proteins (***Fig. 2L*** and ***2M***). These results suggest that hepatic SIRT6 overexpression may protect against liver injury induced by cholestasis, along with preventing oxidative stress, mitochondrial dysfunction, and apoptosis.

### MDL801 activated hepatic SIRT6 to relieve cholestatic liver disease

**Figure 3:**
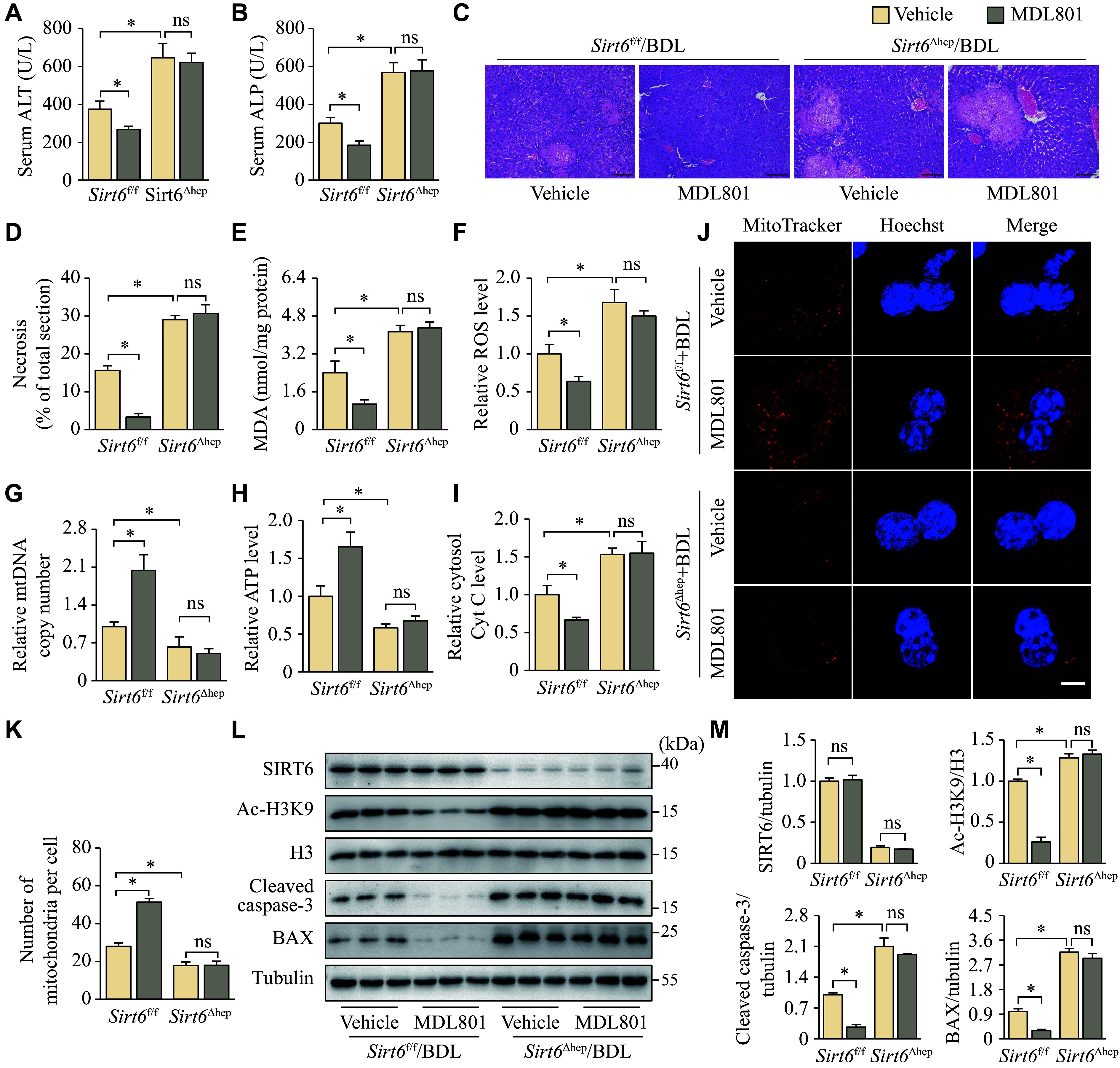
MDL801 relieved cholestatic liver disease in a SIRT6-dependent manner. *Sirt6*^f/f^ and *Sirt6*^Δhep^ mice underwent BDL and were maintained for six days after surgery. Mice injected intraperitoneally with vehicle or MDL801 (100 mg/kg) one day before BDL and daily after BDL were randomly divided into four groups: *Sirt6*^f/f^ + Vehicle, *Sirt6*^f/f^ + MDL801, *Sirt6*^Δhep^ + Vehicle, and *Sirt6*^Δhep^ + MDL801 (*n* = 7 in each group). A and B: The serum was harvested for ALT (A) and ALP (B) activity assays. C: The liver tissues were fixed in 4% paraformaldehyde and embedded in paraffin for hematoxylin-eosin staining. Scale bar, 100 μm. D: The ratio of necrotic area to total liver section area was calculated by ImageJ. E: The liver tissues were homogenized to assay the MDA levels. F: The liver tissues were homogenized to assay the ROS levels, which were normalized to the group *Sirt6*^f/f^ + Vehicle. G: mtDNA was extracted from the livers for quantitative PCR. *Cox1* was determined as an mtDNA marker with *36B4* as the internal standard. H: The liver tissues were homogenized to assay the ATP levels, which were normalized to the group *Sirt6*^f/f^ + Vehicle. I: Liver cytosol was extracted to assay cytosolic Cyt C levels, which were normalized to the group *Sirt6*^f/f^ + Vehicle. J: Hepatocytes isolated from mice underwent fluorescence MitoTracker Red staining (*n* = 3). Scale bar, 10 μm. K: The number of mitochondria was quantified from three different hepatocytes. L: The protein levels of SIRT6, Ac-H3K9, H3, cleaved caspase-3, and BAX in the liver tissues were determined by Western blotting (*n* = 3). Tubulin served as the internal control. M: The quantification of bands of SIRT6, Ac-H3K9, H3, cleaved caspase-3, and BAX. Data are presented as mean ± standard error of the mean and analyzed using two-way ANOVA followed by Tukey's honestly significant difference tests. ^*^P < 0.05. Abbreviations: BDL, bile duct ligation; ALT, aminotransferase; ALP, alkaline phosphatase; MDA, malondialdehyde; *Cox1*, cytochrome C oxidase 1; Cyt C, cytochrome C; H3, histone H3; Ac-H3K9, histone H3 acetyl-lysine 9; ns, not significant.

To assess the therapeutic potential of SIRT6 as a target for cholestasis, we used the SIRT6 agonist MDL801 to treat *Sirt6*^f/f^ and *Sirt6*^Δhep^ mice that underwent BDL. Consistent with ***Fig. 1***, *Sirt6*^Δhep^ mice suffered more severe liver damage than *Sirt6*^f/f^ mice (***Fig. 3***). Similar to SIRT6 overexpression, MDL801 effectively counteracted the elevation of serum ALT and ALP levels (***Fig. 3A*** and ***3B***), reduced the extent of hepatic necrosis (***Fig. 3C*** and ***3D***), and inhibited the increase in liver MDA and ROS levels (***Fig. 3E*** and ***3F***) in *Sirt6*^f/f^ mice. It also increased mtDNA copy number and ATP levels (***Fig. 3G*** and ***3H***), prevented the leakage of Cyt C into the hepatic cytoplasm (***Fig. 3I***), reversed the BDL-induced reduction in mitochondrial number (***Fig. 3J*** and ***3K***), and decreased the expression of cleaved caspase-3 and BAX proteins (***Fig. 3L*** and ***3M***). The selective activation of SIRT6 by MDL801 was evidenced by the fact that MDL801 induced a significant decrease in the acetylation levels of histone H3, a substrate for SIRT6, in the livers of *Sirt6*^f/f^ mice, but this effect was not observed in *Sirt6*^Δhep^ mice (***Fig. 3L*** and ***3M***).

Collectively, MDL801 did not exhibit a significant therapeutic response in bile duct-ligated *Sirt6*^Δhep^ mice (***Fig. 3***), implying that its therapeutic efficacy may primarily arise from its targeting of hepatic SIRT6, rather than activation of SIRT6 in other tissues or from non-SIRT6-agonistic effects.

### Scavenging hepatic oxidative stress did not compensate for the exacerbation of cholestatic liver disease caused by *Sirt6* deficiency

**Figure 4:**
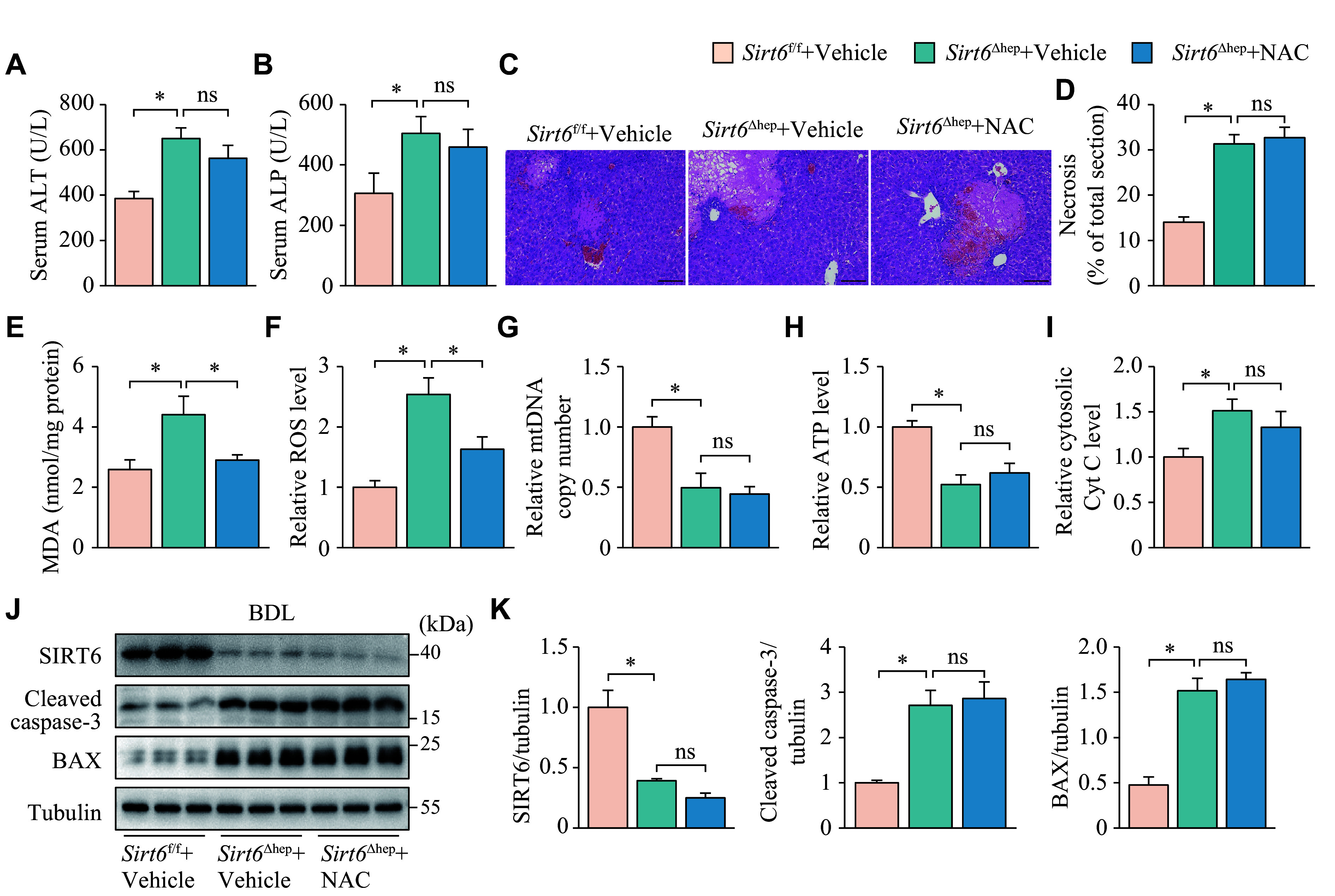
NAC alleviated oxidative stress but not liver injury or mitochondrial dysfunction in mice with cholestatic liver disease. *Sirt6*^f/f^ and *Sirt6*^Δhep^ mice underwent BDL and were maintained for six days after surgery. The mice, treated intraperitoneally with vehicle or NAC (200 mg/kg) one day before BDL and daily after BDL, were randomly divided into three groups: *Sirt6*^f/f^ + Vehicle, *Sirt6*^Δhep^ + Vehicle, and *Sirt6*^Δhep^ + NAC (*n* = 7 in each group). A and B: The serum was collected for ALT (A) and ALP (B) activity assays. C: The liver tissues were fixed in 4% paraformaldehyde and embedded in paraffin for hematoxylin-eosin staining. Scale bar, 100 μm. D: The ratio of necrotic area to total liver section area was calculated by ImageJ. E: The liver tissues were homogenized to assay the MDA levels. F: The liver tissues were homogenized to assay the ROS levels, which were normalized to the group *Sirt6*^f/f^ + Vehicle. G: mtDNA was extracted from the livers for quantitative PCR. *Cox1* was determined as an mtDNA marker with *36B4* as the internal standard. H: The liver tissues were homogenized to assay the ATP levels, which were normalized to the group *Sirt6*^f/f^ + Vehicle. I: Liver cytosol was extracted for assaying cytosolic Cyt C levels, which were normalized to the group *Sirt6*^f/f^ + Vehicle. J: The protein levels of SIRT6, cleaved caspase-3, and BAX in the liver tissues were determined by Western blotting (*n* = 3). Tubulin served as the internal control. K: The quantification of bands of SIRT6, cleaved caspase-3, and BAX. Data are presented as mean ± standard error of the mean and analyzed using one-way ANOVA followed by Tukey's honestly significant difference tests. ^*^P < 0.05. Abbreviations: BDL, bile duct ligation; NAC, N-acetylcysteine; ALT, aminotransferase; ALP, alkaline phosphatase; MDA, malondialdehyde; *Cox1*, cytochrome C oxidase 1; Cyt C, cytochrome C; ns, not significant.

To investigate whether *Sirt6*'s ability to combat oxidative stress contributes to cholestasis treatment, we used a potent antioxidant, NAC, to treat hepatic *Sirt6*-deficient mice that underwent BDL. The results showed that NAC did not reverse the increase in serum ALT and ALP levels (***Fig. 4A*** and ***4B***), nor reduce the extent of hepatic necrosis (***Fig. 4C*** and ***4D***), although it did reduce the elevation of MDA and ROS levels in the livers of *Sirt6*^Δhep^ mice (***Fig. 4E*** and ***4F***). Additionally, NAC failed to increase the copy number of mtDNA and ATP levels (***Fig. 4G*** and ***4H***), prevent the release of Cyt C into the liver cytoplasm (***Fig. 4I***), or decrease the protein levels of cleaved caspase-3 and BAX (***Fig. 4J*** and ***4K***).

**Figure 5:**
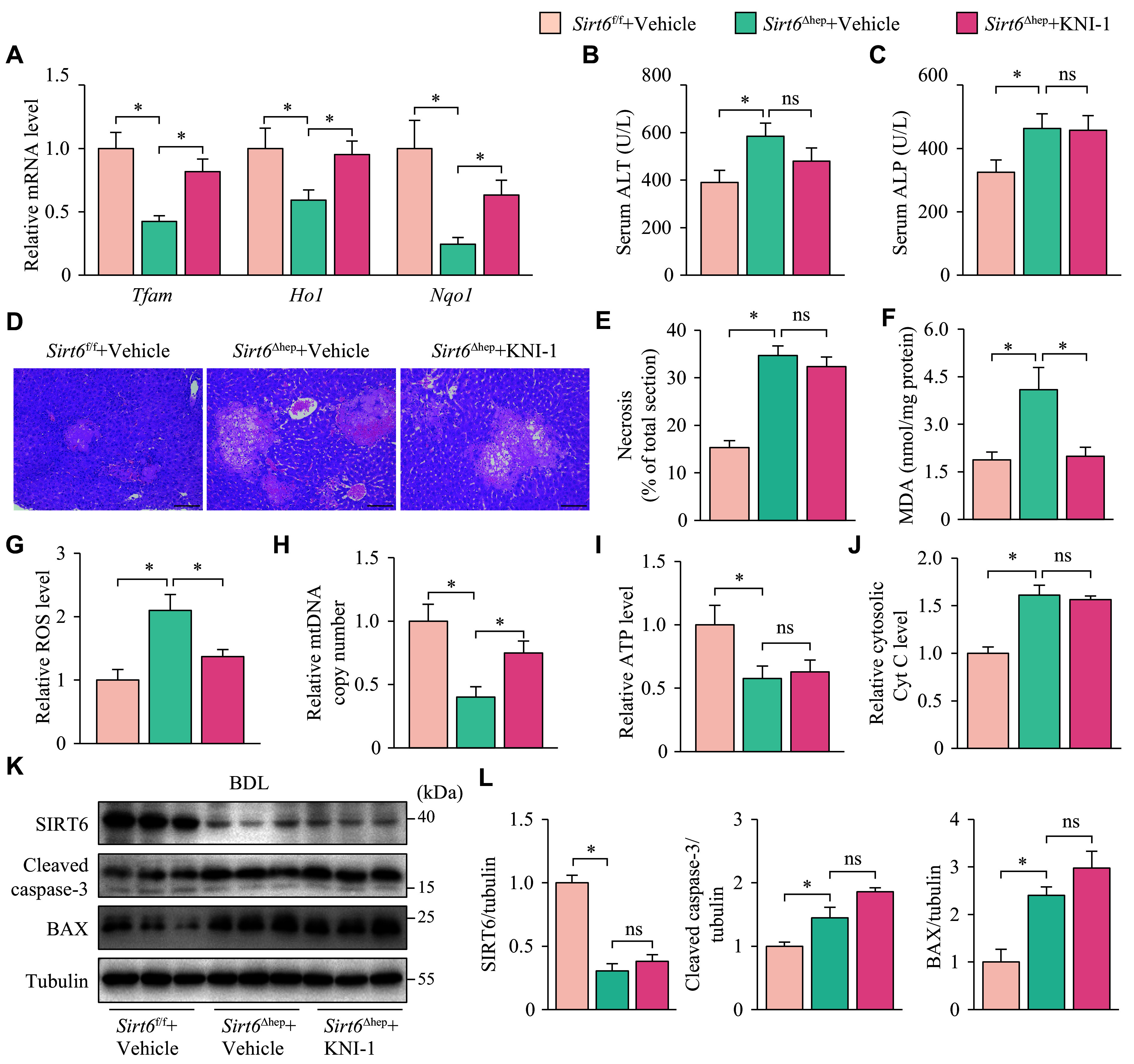
KNI-1 alleviated oxidative stress and promoted mitochondrial biogenesis but did not relieve liver injury or mitochondrial dysfunction in mice with cholestatic liver disease. *Sirt6*^f/f^ and *Sirt6*^Δhep^ mice underwent BDL and were maintained for six days after surgery. The mice, treated intraperitoneally with vehicle or KNI-1 (40 mg/kg) one day before BDL and daily after BDL, were randomly divided into three groups: *Sirt6*^f/f^ + Vehicle, *Sirt6*^Δhep^ + Vehicle, and *Sirt6*^Δhep^ + KNI-1 (*n* = 7 in each group). A: The mRNA levels of *Tfam*, *Ho1*, and *Nqo1* were analyzed by RT-qPCR. B and C: The serum was collected for ALT (B) and ALP (C) activity assays. D: The liver tissues were fixed in 4% paraformaldehyde and embedded in paraffin for hematoxylin-eosin staining. Scale bar, 100 μm. E: The ratio of necrotic area to total liver section area was calculated by ImageJ. F: The liver tissues were homogenized to assay the MDA levels. G: The liver tissues were homogenized to assay the ROS levels, which were normalized to the group *Sirt6*^f/f^ + Vehicle. H: mtDNA was extracted from the livers for quantitative PCR. *Cox1* was determined as an mtDNA marker with *36B4* as the internal standard. I: The liver tissues were homogenized to assay the ATP levels, which were normalized to the group *Sirt6*^f/f^ + Vehicle. J: Liver cytosol was extracted for assaying cytosolic Cyt C levels, which were normalized to the group *Sirt6*^f/f^ + Vehicle. K: The protein levels of SIRT6, cleaved caspase-3, and BAX in the liver tissues were determined by Western blotting (*n* = 3 in each group). Tubulin served as the internal control. L: The quantification of bands of SIRT6, cleaved caspase-3, and BAX. Data are presented as mean ± standard error of the mean and analyzed using one-way ANOVA followed by Tukey's honestly significant difference tests. ^*^P < 0.05. Abbreviations: BDL, bile duct ligation; ALT, aminotransferase; ALP, alkaline phosphatase; MDA, malondialdehyde; *Cox1*, cytochrome C oxidase 1; Cyt C, cytochrome C; ns, not significant.

To avoid the potential insufficiency of NAC in eliminating oxidative stress due to the challenge of maintaining consistent blood concentrations through intermittent administration, we used the NRF2 agonist KNI-1 in *Sirt6*^Δhep^ mice. This treatment led to an increase in the levels of *Ho1* and *Nqo1*, the antioxidant genes that are transcriptional targets of NRF2 in the liver, providing a sustained ability to counteract oxidative stress (***Fig. 5A***). Like NAC, KNI-1 did not decrease the serum ALT and ALP levels (***Fig. 5B*** and ***5C***) or reduce the extent of liver necrosis (***Fig. 5D*** and ***5E***), although it prevented the increase in MDA and ROS levels and partially restored the copy number of mtDNA in the liver of *Sirt6*^Δhep^ mice (***Fig. 5F***–***5H***). Additionally, KNI-1 failed to restore ATP levels (***Fig. 5I***), inhibit the release of Cyt C into the cytoplasm (***Fig. 5J***), or affect the elevated levels of cleaved caspase-3 and BAX proteins (***Fig. 5K*** and ***5L***). These findings indicate that scavenging or inhibiting oxidative stress is not sufficient to counteract the exacerbation of cholestatic liver disease resulting from *Sirt6* deficiency.

### Stimulation of mitochondrial biogenesis did not compensate for the exacerbation of cholestatic liver disease caused by *Sirt6* deficiency

**Figure 6:**
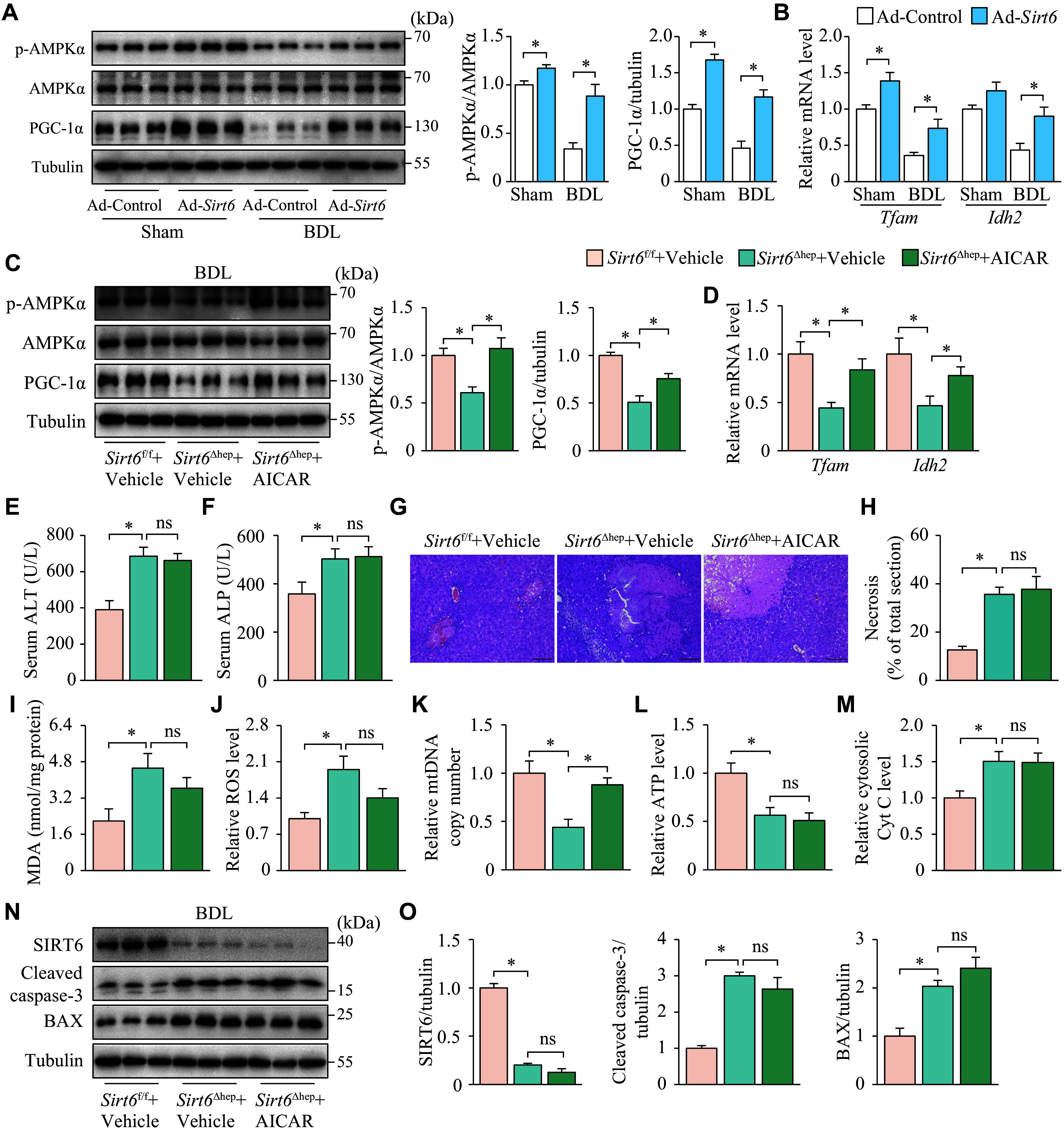
AICAR failed to alleviate cholestatic liver injury, oxidative stress, and mitochondrial dysfunction in mice. A–B: C57BL/6 mice, infected by adenoviral vector expressing SIRT6 (Ad-*Sirt6*) or control vector (Ad-Control), underwent sham or BDL seven days later and were maintained for six days after surgery. All animals were divided into four groups: Ad-Control + Sham, Ad-*Sirt6* + Sham, Ad-Control + BDL, and Ad-*Sirt6* + BDL (*n* = 7 in each group). A: Western blotting analysis of p-AMPK, AMPK, and PGC-1α protein levels in the liver tissues. B: The mRNA levels of *Tfam* and *Idh2* were analyzed by RT-qPCR. C–O: *Sirt6*^f/f^ and *Sirt6*^Δhep^ mice underwent BDL and were maintained for six days after BDL. The mice, treated intraperitoneally with vehicle or AICAR (250 mg/kg) one day before BDL and daily after BDL, were randomly divided into three groups: *Sirt6*^f/f^ + Vehicle, *Sirt6*^Δhep^ + Vehicle, *Sirt6*^Δhep^ + AICAR (*n* = 7 in each group). C: Western blotting analysis of p-AMPK, AMPK, and PGC-1α protein levels in the liver tissues. D: The mRNA levels of *Tfam* and *Idh2* were analyzed by qPCR. E and F: The serum was collected for ALT (E) and ALP (F) activity assays. G: The liver tissues were fixed in 4% paraformaldehyde and embedded in paraffin for hematoxylin-eosin staining. Scale bar, 100 μm. H: The ratio of necrotic area to total liver section area was calculated by ImageJ. I: The liver tissues were homogenized to assay MDA levels. J: The liver tissues were homogenized to assay the ROS levels, which were normalized to the group *Sirt6*^f/f^ + Vehicle. K: mtDNA was extracted from the livers for quantitative PCR. *Cox1* was determined as an mtDNA marker with *36B4* as the internal standard. L: The liver tissues were homogenized to assay the ATP levels, which were normalized to the group *Sirt6*^f/f^ + Vehicle. M: Liver cytosol was extracted for assaying cytosolic Cyt C levels, which were normalized to the group *Sirt6*^f/f^ + Vehicle. N: The protein levels of SIRT6, cleaved caspase-3, and BAX in the liver tissues were evaluated by Western blotting (*n* = 3). Tubulin served as the internal control. O: The quantification of bands of SIRT6, cleaved caspase-3, and BAX. Data are presented as mean ± standard error of the mean and analyzed using two-way (A–B) and one-way ANOVA (C–O) followed by Tukey's honestly significant difference tests. ^*^P < 0.05. Abbreviations: BDL, bile duct ligation; ALT, aminotransferase; ALP, alkaline phosphatase; MDA, malondialdehyde; *Cox1*, cytochrome C oxidase 1; Cyt C, cytochrome C; ns, not significant.

The intracellular accumulation of bile acids may disrupt the structure and function of mitochondria, resulting in apoptosis and necrosis. Therefore, restoring mitochondrial structure by promoting mitochondrial biogenesis may potentially offer an effective approach to reverse cholestatic liver disease. It has been reported that SIRT6 enhances the expression of genes involved in mitochondrial biogenesis, such as *Tfam*, through the AMPK/PGC-1α pathway. This mechanism helps defend biliary endothelial cells against bile acid-induced apoptosis^[17]^. Consistent with this finding, we found that hepatic overexpression of SIRT6 triggered the AMPK/PGC-1α pathway and increased the expression levels of genes related to mitochondrial biogenesis, including *Tfam* and *Idh2* (***Fig. 6A*** and ***6B***). To investigate the role of mitochondrial biogenesis in SIRT6's resistance to cholestatic liver disease, we administered the AMPK agonist AICAR to *Sirt6*^Δhep^ mice, and observed a significant restoration of the AMPK phosphorylation and PGC-1α protein levels that were impaired by *Sirt6* deletion. This intervention also resulted in increased transcript levels of *Tfam* and *Idh2* (***Fig. 6C*** and ***6D***). Despite this, AICAR did not result in any notable improvements in serum ALT and ALP levels, hepatic necrosis area, ATP levels, hepatocyte cytoplasmic Cyt C levels, or cleaved caspase-3 and BAX protein levels, except for the partially recovered mtDNA copy number (***Fig. 6E***–***6O***). The results indicate that enhancing mitochondrial biogenesis through the AMPK/PGC-1α pathway is not a critical requirement for SIRT6 to exert its anticholestatic function.

### SIRT6 primarily reduced bile acid synthesis to alleviate cholestatic liver disease

**Figure 7:**
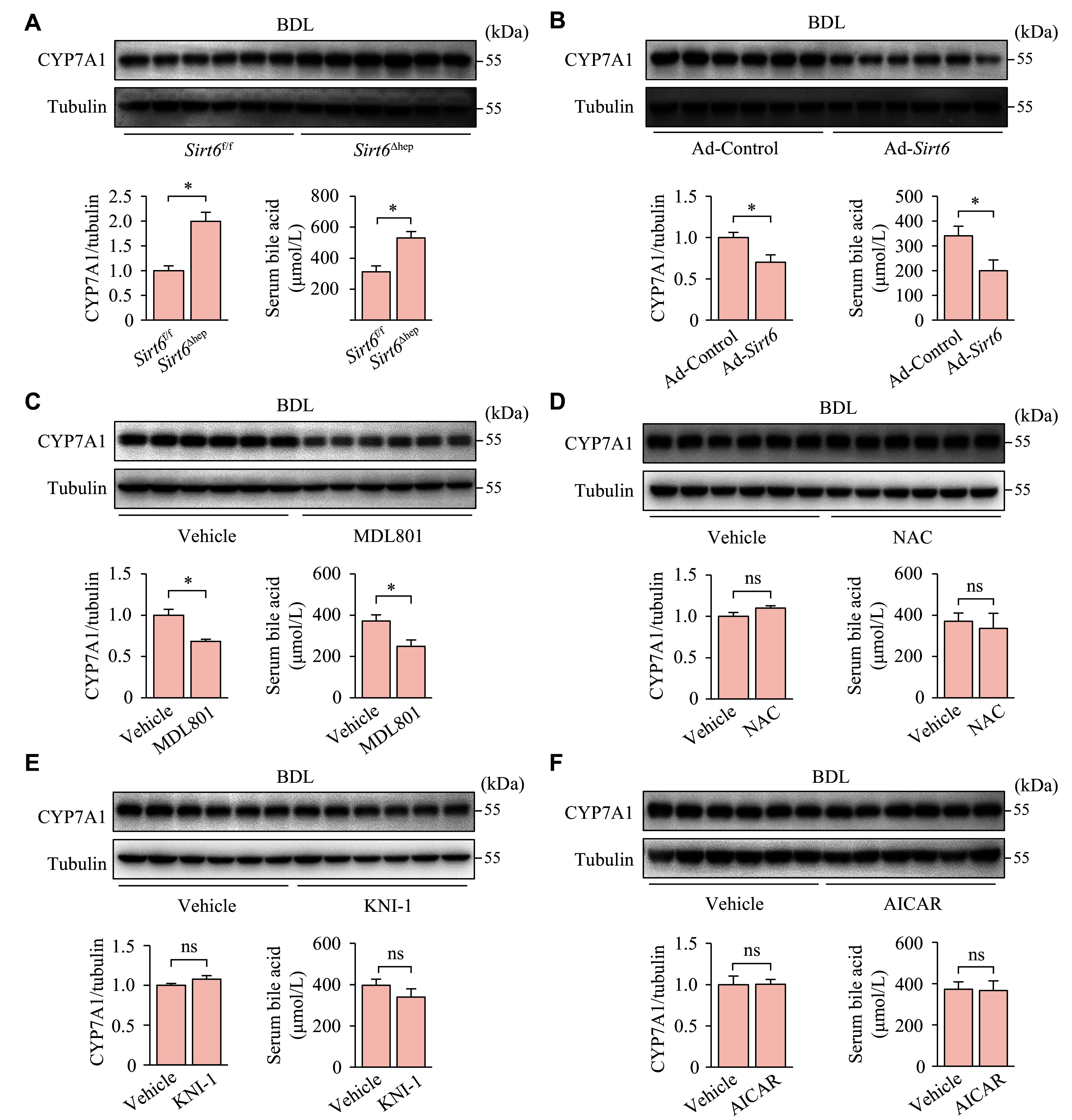
MDL801 rather than KNI-1, AICAR, or NAC reduced bile acid synthesis in mice with cholestatic liver disease. A: *Sirt6*^f/f^ and *Sirt6*^Δhep^ mice underwent BDL and were maintained for six days (*n* = 6 in each group) after surgery. B: C57BL/6 mice, infected with an adenoviral vector expressing SIRT6 (Ad-*Sirt6*) or control vector (Ad-Control), underwent BDL seven days later, and were maintained for six days (*n* = 6 in each group). C: *Sirt6*^f/f^ mice underwent BDL and were maintained for six days. The mice, treated intraperitoneally with vehicle or MDL801 (100 mg/kg) one day before BDL and daily after BDL, were randomly divided into two groups: *Sirt6*^f/f^ + Vehicle and *Sirt6*^f/f^ + MDL801 (*n* = 6 in each group). D: *Sirt6*^f/f^ mice underwent BDL and were maintained for six days. The mice, treated intraperitoneally with vehicle or NAC (200 mg/kg) one day before BDL and daily after BDL, were randomly divided into two groups: *Sirt6*^f/f^ + Vehicle and *Sirt6*^f/f^ + NAC (*n* = 6 in each group). E: *Sirt6*^f/f^ mice underwent BDL and were maintained for six days. The mice, treated intraperitoneally with vehicle or KNI-1 (40 mg/kg) one day before BDL and daily after BDL, were randomly divided into two groups: *Sirt6*^f/f^ + Vehicle and *Sirt6*^f/f^ + KNI-1 (*n* = 7 in each group). F: *Sirt6*^f/f^ mice underwent BDL and were maintained for six days. The mice, treated intraperitoneally with vehicle or AICAR (250 mg/kg) one day before BDL and daily after BDL, were randomly divided into two groups: *Sirt6*^f/f^ + Vehicle and *Sirt6*^f/f^ + AICAR (*n* = 6 in each group). The protein levels of CYP7A1 in the liver tissues were determined by Western blotting. Tubulin served as the internal control. The serum was collected for bile acid level assay. Data are presented as mean ± standard error of the mean and analyzed using Student's *t*-test. ^*^P < 0.05. Abbreviations: BDL, bile duct ligation; NAC, N-acetylcysteine; KNI-1, KEAP1-NRF2-IN-1; AICAR, acadesine; ns, not significant.

Reducing bile acid production is a successful approach to ameliorating liver damage caused by cholestasis. The serum levels of bile acids may be elevated because of the release of bile acids into the bloodstream as a result of impaired enterohepatic circulation or the overproduction of bile acids. In the mice with BDL, *Sirt6* deficiency in the liver led to a notable increase in the levels of serum bile acids and hepatic CYP7A1, a crucial enzyme responsible for synthesizing bile acids (***Fig. 7A***). In contrast, elevated expression of hepatic SIRT6 or MDL801, a SIRT6 agonist, significantly reduced the CYP7A1 expression and serum bile acid levels (***Fig. 7B*** and ***7C***). This suggests that SIRT6 reduces bile acid synthesis by inhibiting the expression of CYP7A1, thereby alleviating cholestasis. We also tested the effect of KNI-1, AICAR, and NAC, which are compounds known to affect mitochondrial biogenesis or oxidative stress. However, none of them inhibited the expression of CYP7A1 or reduced the concentration of bile acids in the bloodstream (***Fig. 7D***–***7F***).

**Figure 8:**
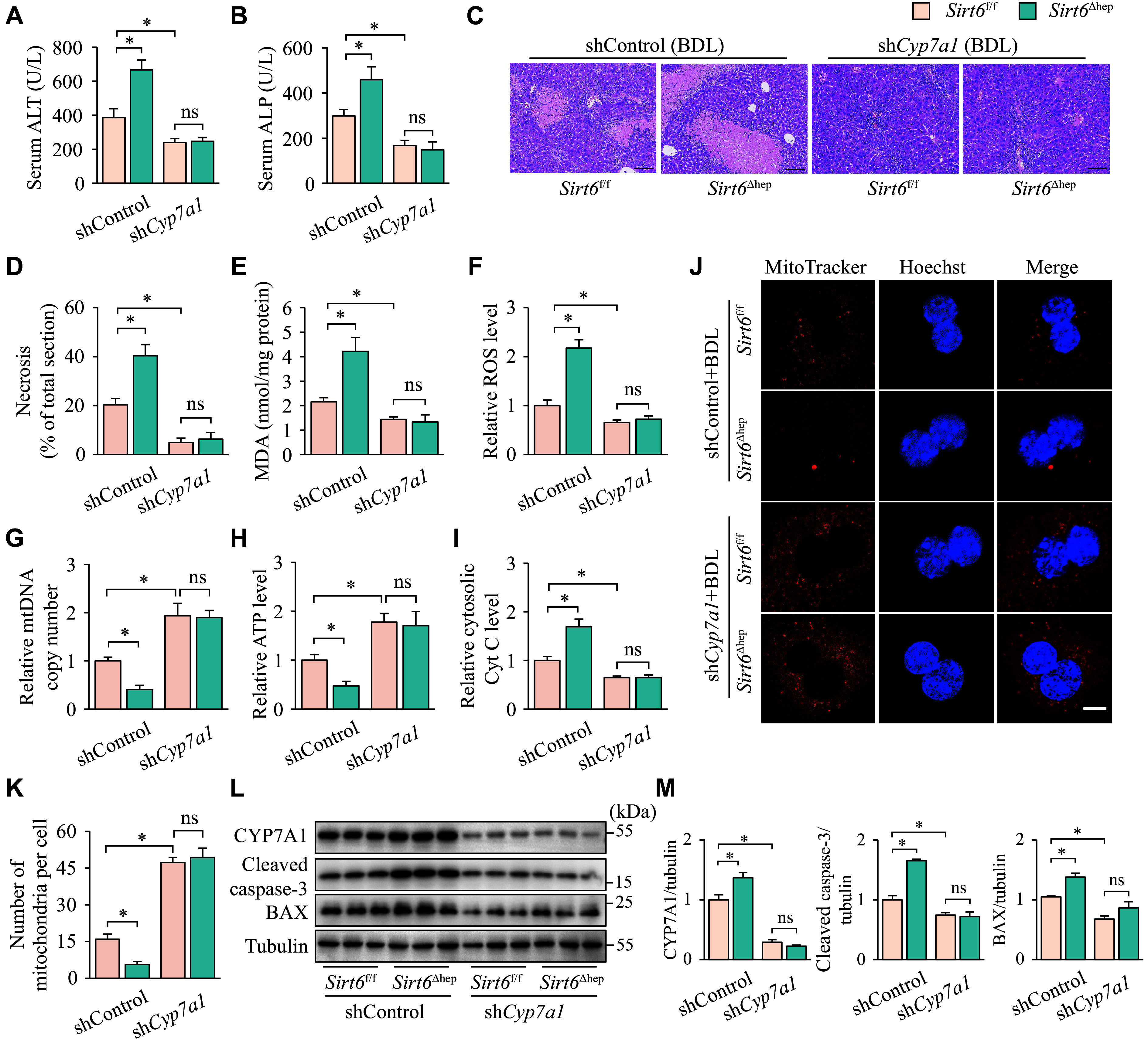
Hepatic knockdown of *Cyp7a1* counteracted cholestatic liver injury caused by hepatic BDL and *Sirt6* deficiency. *Sirt6*^f/f^ or *Sirt6*^Δhep^ mice, infected either with an adeno-associated virus knocking down *Cyp7a1* (sh*Cyp7a1*) or a control vector (shControl), underwent BDL three weeks later and were maintained for six days after surgery. Mice were divided into four groups: *Sirt6*^f/f^ + shControl, *Sirt6*^Δhep^ + shControl, *Sirt6*^f/f^ + sh*Cyp7a1*, and *Sirt6*^Δhep^ + sh*Cyp7a1* (*n* = 7 in each group). A and B: The serum was collected for ALT (A) and ALP (B) activity assays. C: The liver tissues were fixed in 4% paraformaldehyde and embedded in paraffin for hematoxylin-eosin staining. Scale bar, 100 μm. D: The ratio of necrotic area to total liver section area was calculated by ImageJ. E: The liver tissues were homogenized to assay MDA levels. F: The liver tissues were homogenized to assay ROS levels, and the levels in each group were normalized to the group *Sirt6*^f/f^ + shControl. G: mtDNA was extracted from livers for quantitative PCR. *Cox1* was determined as an mtDNA marker with *36B4* as the internal standard. H: The liver tissues were homogenized to assay the ATP levels, which were normalized to the group *Sirt6*^f/f^ + shControl. I: Liver cytosol was extracted for assaying cytosolic Cyt C levels, which were normalized to the group *Sirt6*^f/f^ + shControl. J: Hepatocytes isolated from mice underwent fluorescence MitoTracker Red staining (*n* = 3). Scale bar, 10 μm. K: The number of mitochondria was quantified from three different hepatocytes. L: The protein levels of SIRT6, cleaved caspase-3, and BAX in the liver tissues were determined by Western blotting (*n* = 3). Tubulin served as the internal control. M: The quantification of bands of SIRT6, cleaved caspase-3, and BAX. Data are presented as mean ± standard error of the mean and analyzed using two-way ANOVA followed by Tukey's honestly significant difference tests. ^*^P < 0.05. Abbreviations: BDL, bile duct ligation; ALT, aminotransferase; ALP, alkaline phosphatase; MDA, malondialdehyde; *Cox1*, cytochrome C oxidase 1; Cyt C, cytochrome C; ns, not significant.

To further validate the importance of down-regulating CYP7A1 in the anticholestatic effects of SIRT6, we used adeno-associated viral vectors to specifically knock down *Cyp7a1* in the livers of *Sirt6*^f/f^ and *Sirt6*^Δhep^ mice with BDL. Consistent with ***Fig. 1***, *Sirt6*^Δhep^ mice had more severe cholestasis than *Sirt6*^f/f^ mice when receiving shControl (***Fig. 8***). However, hepatic *Cyp7a1* knockdown significantly relieved BDL-induced cholestasis in *Sirt6*^f/f^ mice. *Sirt6*^f/f^ mice receiving sh*Cyp7a1* had lower serum ALT and ALP levels (***Fig. 8A*** and ***8B***), less hepatic necrosis (***Fig. 8C*** and ***8D***), lower liver MDA and ROS levels (***Fig. 8E*** and ***8F***), more mtDNA copy numbers (***Fig. 8G***), higher ATP levels (***Fig. 8H***), less Cyt C entering the hepatocyte cytoplasm (***Fig. 8I***), more mitochondrial content in hepatocytes (***Fig. 8J*** and ***8K***), and lower levels of cleaved caspase-3 and BAX proteins (***Fig. 8L*** and ***8M***). Furthermore, hepatic *Cyp7a1* knockdown smoothed out the differences between *Sirt6*^f/f^ and *Sirt6*^Δhep^ mice based on these metrics (***Fig. 8***).

## Discussion

The present study demonstrates that SIRT6 acts as a protective factor against BDL-induced cholestatic liver disease. Based on existing research and experimental findings, we propose that SIRT6 may potentially deliver its therapeutic effects by reducing oxidative stress, promoting mitochondrial biogenesis, or suppressing the production of bile acids. By analyzing the three pathways, we discovered that the blocking of bile acid production might be the primary mechanism underlying the anticholestatic properties of SIRT6.

Bile acids interfere with the functioning of large conductance channels located in the mitochondrial inner membrane, specifically those sensitive to cyclosporine A and trifluoperazine. This disruption triggers the opening of the mitochondrial permeability transition pore, resulting in the collapse of the mitochondrial inner transmembrane potential, expansion of the matrix space, and eventual rupture of the outer membrane. The collapse of the mitochondrial transmembrane potential disconnects the respiratory chain, preventing the production of ATP and promoting the generation of ROS. When the mitochondrial membrane breaks, it results in the release of mitochondrial proteins like Cyt C. Furthermore, bile acids induce conformational alterations in a pro-apoptotic protein, BAX, prompting its shift from the cytoplasm to the mitochondria, thus initiating the release of Cyt C. When Cyt C is released, cell death may occur either through an apoptotic mechanism involving caspase activation or through necrosis arising from the disruption of electron transport^[31]^.

When ROS levels increase, cells experience oxidative stress, leading to damage to DNA, proteins, and lipids, ultimately affecting the integrity of cell membranes and inducing apoptosis^[30]^. However, our experiments showed that treating cholestasis-induced liver injury with antioxidant methods alone did not lead to a reversal of this condition. It is speculated that oxidative stress, as a downstream event of compromised mitochondrial membrane potential and structural anisotropy, even when diminished, cannot halt the upstream mitochondrial damage or mitochondrial damage-triggered apoptosis and necrosis.

Mitochondrial biogenesis of hepatocytes is severely impaired in cholestatic liver disease. PGC-1α, the key transcriptional activator in mitochondrial biogenesis^[19]^, acts to increase the production of mitochondrial components such as IDH2, cytochrome C oxidase subunit Ⅳ (COX Ⅳ), and Cyt C. Additionally, PGC-1α enhances the expression of the transcription factor NRF2, triggering the transcription of *Tfam*^[17,32]^. TFAM plays a pivotal role by binding to mtDNA, contributing to its stabilization, replication, and transcription of mtDNA^[33–34]^. It's worth noting that mtDNA encodes crucial subunits of the respiratory chain complex, which play a pivotal role in the process of mitochondrial biogenesis^[4–5]^. When bile acids accumulate, they block the expression of PGC-1α and its downstream genes, including *Nrf2*, *Tfam*, and various mitochondrial proteins^[35]^. Therefore, increasing levels of SIRT6 and using a SIRT6 agonist might reverse this inhibition of those mitochondrial biogenesis-related genes. To investigate whether mitochondrial biogenesis affects SIRT6's resistance to cholestatic liver disease, we administered AICAR to liver-specific *Sirt6* knockout mice to activate the AMPK/PGC-1α pathway, or used KNI-1 to activate NRF2. Although these approaches increased the expression levels of genes related to mitochondrial biogenesis, they did not counteract the exacerbation of liver damage following the *Sirt6* knockdown. Therefore, mitochondrial biogenesis may not be the main pathway through which SIRT6 mitigates cholestatic liver disease. The accumulation of bile acids in liver cells likely causes continuous damage to newly developed mitochondria, so increasing mitochondrial production does not improve overall mitochondrial function.

It has been reported that SIRT6 inhibits *Cyp7a1* transcription, thereby decreasing bile acid synthesis and alleviating liver injury associated with cholestasis^[20]^. Considering that neither oxidative stress nor mitochondrial biogenesis serves as a pathway for SIRT6 in combating cholestatic liver disease, we theorized that the inhibition of bile acid synthesis might be the key mechanism underlying SIRT6's therapeutic function. Supporting this, the SIRT6 agonist MDL801, as opposed to NAC, KNI-1, or AICAR, reduced the expression level of hepatic CYP7A1 and the concentration of bile acids in the serum in BDL mice. Additionally, the knockdown of hepatic CYP7A1, which serves as a key enzyme in the classical synthesis pathway for bile acids^[36]^, significantly alleviated BDL-induced cholestasis and counteracted the adverse effects of *Sirt6* deletion.

Impeding the synthesis of bile acids by downregulation of CYP7A1 has become a widely adopted approach in the treatment of cholestatic liver diseases. Taking obeticholic acid, cilofexor, and tropifexor as examples, these steroidal and non-steroidal farnesoid X receptor (FXR) agonists activate the FXR/small heterodimer partner (SHP) pathway and thereby inhibit hepatocyte nuclear factor 4α (HNF4α)-mediated transcription of *Cyp7a1*^[37–39]^. Similarly, alafermin, a fibroblast growth factor 19 (FGF19) analog, disrupts transcription factor EB (TFEB)-mediated transcription of *Cyp7a1* through the mechanistic target of rapamycin/extracellular signal-regulated kinase pathway^[37,40]^. Fibrates stimulate peroxisome proliferator-activated receptor (PPARα), resulting in the transcriptional repression of *Cyp7a1*^[37,41]^. Activation of SIRT6 reduces *Cyp7a1*'s transcription by inhibiting estrogen-related receptors (ERRγ)^[20]^, which is a completely novel regulatory pathway. Therefore, SIRT6 agonists may be highly synergistic with the above drugs, allowing for lower drug dosages, thereby reducing adverse effects, such as dyslipidemia and itchiness from FXR agonists, carcinogenic risks from FGF19 analogs, and myalgia as well as rhabdomyolysis from PPARα agonists^[37]^.

The intestinal flora also plays a key role in bile acid metabolism. Gut microbiota with bile salt hydrolase activity can remove the glycine or taurine from conjugated bile acids to increase their hydrophobicity, thereby facilitating the fecal elimination of bile acids^[42]^. This function is synergistic with SIRT6 agonists, and potentially beneficial for resistance to bile toxicity and cholestasis. Notably, bile acids play a role in shaping the gut microbiome by promoting the growth of bile-acid-metabolizing bacteria and inhibiting the development of other bacteria that are sensitive to bile^[42]^. Thus, cholestasis or SIRT6 agonists, which likely interfere with the flow of bile acids into the small intestine, may reduce the population of bile acid-metabolizing bacteria but lead to an overgrowth of other harmful bacteria in the gut. Therefore, during the administration of SIRT6 agonists for cholestasis treatment, it is beneficial to moderately increase the population of bile-acid-metabolizing bacteria and maintain intestinal flora balance, as this may increase the efficacy of the agonists and lessen potential adverse effects.

In conclusion, SIRT6 reduces hepatic bile acid production by inhibiting the expression of CYP7A1, a key enzyme in bile acid synthesis, and alleviates the toxic stress of bile acids on hepatocytes. This regulatory mechanism is the key to the amelioration of cholestatic liver disease by SIRT6, as compared to the enhancement of antioxidant gene expression or the promotion of mitochondrial biogenesis.

## SUPPLEMENTARY DATA

Supplementary data to this article can be found online.

## References

[1] (2021). The role of bile acids in cholestatic liver injury. Ann Transl Med.

[2] (2013). Oxidative stress plays a major role in chlorpromazine-induced cholestasis in human HepaRG cells. Hepatology.

[3] (2003). Mitochondrially mediated synergistic cell killing by bile acids. Biochim Biophys Acta Mol Basis Dis.

[4] (2005). Mitochondrial DNA mutations, oxidative stress, and apoptosis in mammalian aging. Science.

[5] (2008). Mitochondrial DNA damage and repair in neurodegenerative disorders. DNA Repair (Amst).

[6] (1999). Mitochondrial diseases in man and mouse. Science.

[7] (2024). Heritable chronic cholestatic liver diseases: A review. J Clin Transl Hepatol.

[8] (2024). Progress in the management of patients with cholestatic liver disease: Where are we and where are we going?. J Clin Transl Hepatol.

[9] (2015). Primary biliary cirrhosis. Lancet.

[10] (2013). SIRT6 regulates TNF-α secretion through hydrolysis of long-chain fatty acyl lysine. Nature.

[11] (2005). Mouse Sir2 homolog SIRT6 is a nuclear ADP-ribosyltransferase. J Biol Chem.

[12] (2021). Emerging roles of SIRT6 in human diseases and its modulators. Med Res Rev.

[13] (2017). Hepatocyte-specific sirtuin 6 deletion predisposes to nonalcoholic steatohepatitis by up-regulation of Bach1, an Nrf2 repressor. FASEB J.

[14] (2021). SIRT6 as a key event linking P53 and NRF2 counteracts APAP-induced hepatotoxicity through inhibiting oxidative stress and promoting hepatocyte proliferation. Acta Pharm Sin B.

[15] (2023). Sirt6 mediates antioxidative functions by increasing Nrf2 abundance. Exp Cell Res.

[16] (2022). Hepatic SIRT6 modulates transcriptional activities of FXR to alleviate acetaminophen-induced hepatotoxicity. Cell Mol Gastroenterol Hepatol.

[17] (2020). Sirt6 opposes glycochenodeoxycholate-induced apoptosis of biliary epithelial cells through the AMPK/PGC-1α pathway. Cell Biosci.

[18] (2021). Melatonin attenuates diabetic cardiomyopathy and reduces myocardial vulnerability to ischemia-reperfusion injury by improving mitochondrial quality control: Role of SIRT6. J Pineal Res.

[19] (2023). SIRT6 is a key regulator of mitochondrial function in the brain. Cell Death Dis.

[20] (2020). ERRγ suppression by Sirt6 alleviates cholestatic liver injury and fibrosis. JCI Insight.

[21] (2024). Tannic acid- and N-acetylcysteine-chitosan-modified magnetic nanoparticles reduce hepatic oxidative stress in prediabetic rats. Colloids Surf B Biointerfaces.

[22] (2019). Discovery of a potent kelch-like ECH-associated protein 1-nuclear factor erythroid 2-related factor 2 (Keap1-Nrf2) protein-protein interaction inhibitor with natural proline structure as a cytoprotective agent against acetaminophen-induced hepatotoxicity. J Med Chem.

[23] (2022). AICAR stimulates mitochondrial biogenesis and BCAA catabolic enzyme expression in C2C12 myotubes. Biochimie.

[24] (2019). Sirt6 deletion in hepatocytes increases insulin sensitivity of female mice by enhancing ERα expression. J Cell Physiol.

[25] (2012). Effect of bile duct ligation on bile acid composition in mouse serum and liver. Liver Int.

[26] (2018). Combination of coenzyme Q_10_ intake and moderate physical activity counteracts mitochondrial dysfunctions in a SAMP8 mouse model. Oxid Med Cell Longev.

[27] (2012). NF-κB-inducing kinase (NIK) promotes hyperglycemia and glucose intolerance in obesity by augmenting glucagon action. Nat Med.

[28] (2022). Ginsenoside Rb1 inhibits astrocyte activation and promotes transfer of astrocytic mitochondria to neurons against ischemic stroke. Redox Biol.

[29] (2011). Mitochondrial biogenesis fails in secondary biliary cirrhosis in rats leading to mitochondrial DNA depletion and deletions. Am J Physiol Gastrointest Liver Physiol.

[30] (2021). Redox-dependent effects in the physiopathological role of bile acids. Oxid Med Cell Longev.

[31] (2000). Mitochondrial membrane perturbations in cholestasis. J Hepatol.

[32] (2023). Induction of lysosomal and mitochondrial biogenesis by AMPK phosphorylation of FNIP1. Science.

[33] (2011). Mitochondrial genome-maintaining activity of mouse mitochondrial transcription factor A and its transcript isoform in *Saccharomyces cerevisiae*. Gene.

[34] (2012). Mitochondrial transcription factor A regulates mitochondrial transcription initiation, DNA packaging, and genome copy number. Biochim Biophys Acta Gene Regul Mech.

[35] (2012). Damage to mtDNA in liver injury of patients with extrahepatic cholestasis: The protective effects of mitochondrial transcription factor A. Free Radic Biol Med.

[36] (2016). Cholesterol 7α-hydroxylase protects the liver from inflammation and fibrosis by maintaining cholesterol homeostasis. J Lipid Res.

[37] (2023). Primary biliary cholangitis as a roadmap for the development of novel treatments for cholestatic liver diseases. J Hepatol.

[38] (2010). Transcriptional corepressor SHP recruits SIRT1 histone deacetylase to inhibit LRH-1 transactivation. Nucleic Acids Res.

[39] (2012). Nuclear receptors HNF4α and LRH-1 cooperate in regulating *Cyp7a1 in vivo*. J Biol Chem.

[40] (2020). An FGF15/19-TFEB regulatory loop controls hepatic cholesterol and bile acid homeostasis. Nat Commun.

[41] (2004). The inhibition of the human cholesterol 7α-hydroxylase gene (*CYP7A1*) promoter by fibrates in cultured cells is mediated *via* the liver x receptor α and peroxisome proliferator-activated receptor α heterodimer. Nucleic Acids Res.

[42] (2016). Intestinal crosstalk between bile acids and microbiota and its impact on host metabolism. Cell Metab.

