# Supplementary material for: Hepatic SIRT6 protects against cholestatic liver disease primarily via inhibiting bile acid synthesis
Source: J Biomed Res. 2024 Dec 2;39(4):340–55. doi: 10.7555/JBR.38.20240172 (PMC12336411; doi:10.7555/JBR.38.20240172)
Supplement: Supplementary file 1 — Supplementary data to this article can be found online. [file jbr-39-4-340-Supplementary.pdf]

Available online at www.jbr-pub.org.cn

Open Access at PubMed Central

Journal of Biomedical Research, 2025 39(4): 1–2

*Supplementary Data*

JBR

# Hepatic SIRT6 protects against cholestatic liver disease primarily *via* inhibiting bile acid synthesis

Wen Zhang[1], Jiahui Wang[1], Luyao Yang[1], Yuyun Shao[2], Hongjun Peng[3,✉], Longfeng Jiang[4,✉], Liang Sheng[1,✉]

[1]*Department of Pharmacology, School of Basic Medical Sciences, Nanjing Medical University, Nanjing, Jiangsu 211166, China;*
[2]*Endoscopy Center, the First Affiliated Hospital of Nanjing Medical University, Nanjing, Jiangsu 210029, China;*
[3]*Department of Pediatrics, Nanjing Drum Tower Hospital, Clinical College of Nanjing Medical University, Nanjing, Jiangsu 210008, China;*
[4]*Department of Infectious Diseases, the First Affiliated Hospital of Nanjing Medical University, Nanjing, Jiangsu 210029, China.*

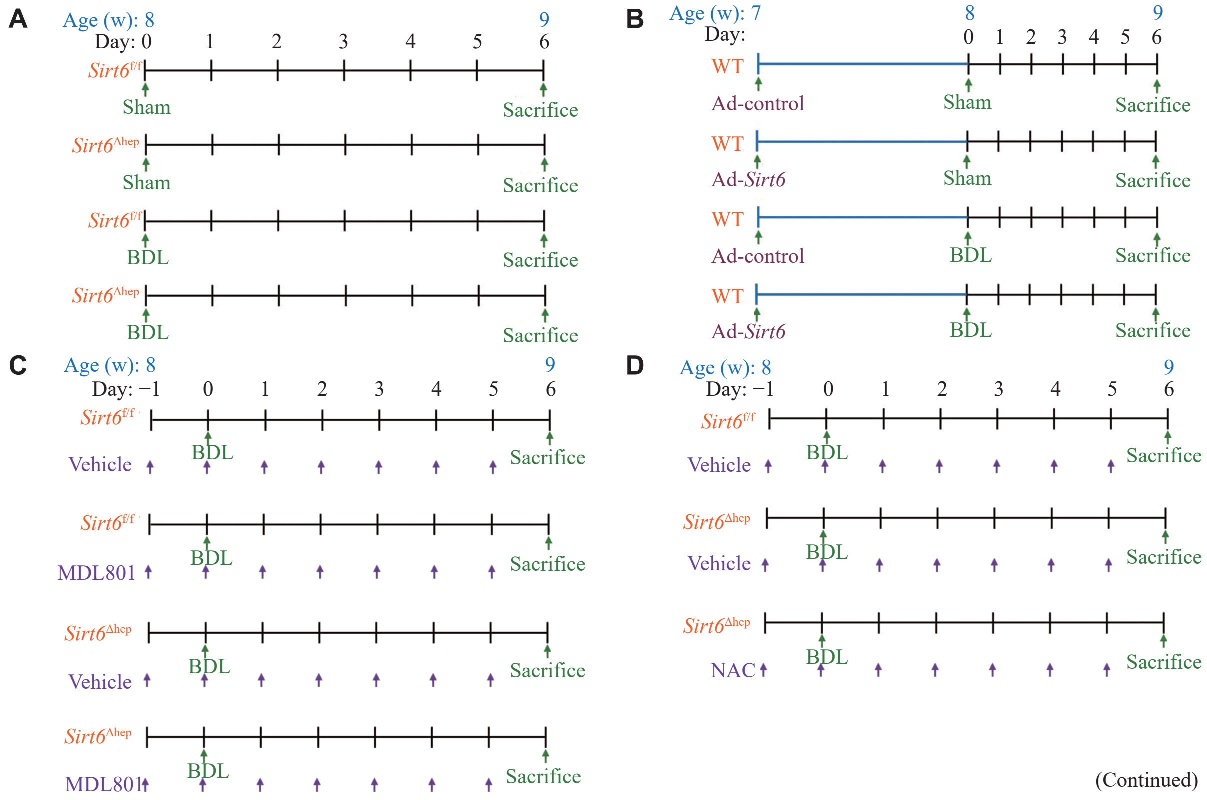


(Continued)

✉Corresponding authors: Hongjun Peng, Department of Pediatrics, Nanjing Drum Tower Hospital, Clinical College of Nanjing Medical University, 321 Zhongshan Road, Nanjing, Jiangsu 210008, China. E-mail: hjpeng@njglyy.com; Longfeng Jiang, Department of Infectious Diseases, the First Affiliated Hospital of Nanjing Medical University, 300 Guangzhou Road, Nanjing, Jiangsu 210029, China. E-mail: longfengjiang@njmu.edu.cn; Liang Sheng, Department of Pharmacology, School of Basic Medical Sciences, Nanjing Medical University, 101 Longmian Avenue, Nanjing, Jiangsu 211166, China. E-mail: lgsheng@njmu.edu.cn.

Received: 10 June 2024; Revised: 23 November 2024; Accepted: 25 November 2024; Published online: 02 December 2024

CLC number: R575.7, Document code: A

The authors reported no conflict of interests.

This is an open access article under the Creative Commons Attribution (CC BY 4.0) license, which permits others to distribute, remix, adapt and build upon this work, for commercial use, provided the original work is properly cited.

(Continued)

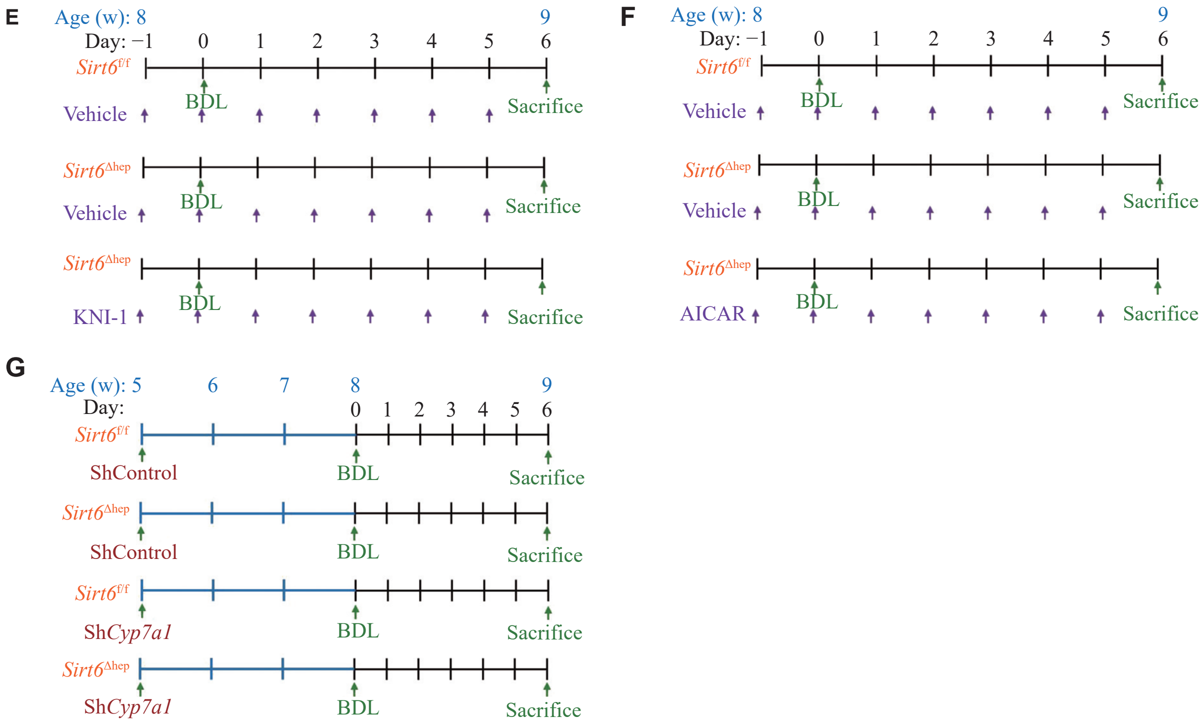


***Supplementary Fig. 1*** **The timeline of animal experiments.** A: *Sirt6*$^{f/f}$ and *Sirt6*$^{\Delta hep}$ mice, male, eight weeks old, underwent sham or BDL and were maintained for six days before sacrifice. The mice were divided into four groups: *Sirt6*$^{f/f}$ + Sham, *Sirt6*$^{\Delta hep}$ + Sham, *Sirt6*$^{f/f}$ + BDL, and *Sirt6*$^{\Delta hep}$ + BDL ($n$ = 7 in each group). B: C57BL/6 mice, male, seven weeks old, were infected with Ad-*Sirt6* or Ad-Control. After seven days, the mice underwent sham or BDL and were maintained for six days. The mice were divided into four groups: Sham + Ad-Control, Sham + Ad-*Sirt6*, BDL + Ad-Control, and BDL + Ad-*Sirt6* ($n$ = 7 in each group). C: *Sirt6*$^{f/f}$ and *Sirt6*$^{\Delta hep}$ mice, male, eight weeks old, underwent BDL and were maintained for six days. The mice, injected intraperitoneally with vehicle or MDL801 (100 mg/kg) one day before BDL and daily after BDL, were randomly divided into four groups: *Sirt6*$^{f/f}$ + Vehicle, *Sirt6*$^{f/f}$ + MDL-801, *Sirt6*$^{\Delta hep}$ + Vehicle, and *Sirt6*$^{\Delta hep}$ + MDL-801 ($n$ = 7 in each group). D: *Sirt6*$^{f/f}$ and *Sirt6*$^{\Delta hep}$ mice, male, eight weeks old, underwent BDL and were maintained for six days. The mice, injected intraperitoneally with vehicle or NAC (200 mg/kg) one day before BDL and daily after BDL, were randomly divided into three groups: *Sirt6*$^{f/f}$ + Vehicle, *Sirt6*$^{\Delta hep}$ + Vehicle, and *Sirt6*$^{\Delta hep}$ + NAC ($n$ = 7 in each group). E: *Sirt6*$^{f/f}$ and *Sirt6*$^{\Delta hep}$ mice, male, eight weeks old, underwent BDL and were maintained for six days. The mice, injected intraperitoneally with vehicle or KNI-1 (40 mg/kg) one day before BDL and daily after BDL, were randomly divided into three groups: *Sirt6*$^{f/f}$ + Vehicle, *Sirt6*$^{\Delta hep}$ + Vehicle, and *Sirt6*$^{\Delta hep}$ + KNI-1 ($n$ = 7 in each group). F: *Sirt6*$^{f/f}$ and *Sirt6*$^{\Delta hep}$ mice, male, eight weeks old, underwent BDL and were maintained for six days. The mice, injected intraperitoneally with vehicle or AICAR (250 mg/kg) one day before BDL and daily after BDL, were randomly divided into three groups: *Sirt6*$^{f/f}$ + Vehicle, *Sirt6*$^{\Delta hep}$ + Vehicle, and *Sirt6*$^{\Delta hep}$ + AICAR ($n$ = 7 in each group). G: *Sirt6*$^{f/f}$ and *Sirt6*$^{\Delta hep}$ mice, male, five weeks old, were infected with adeno-associated virus expressing sh*Cyp7a1* or shControl. After 21 days, the mice underwent BDL and were maintained for six days. The mice were divided into four groups: *Sirt6*$^{f/f}$ + shControl, *Sirt6*$^{\Delta hep}$ + shControl, *Sirt6*$^{f/f}$ + sh*Cyp7a1*, and *Sirt6*$^{\Delta hep}$ + sh*Cyp7a1* ($n$ = 7 in each group). Abbreviations: BDL, bile duct ligation; NAC, N-acetylcysteine; KNI-1, KEAP1-NRF2-IN-1; AICAR, acadesine.
